# Screening of Chemical Residues and Antimicrobial Resistance in Dehydrated Chicken Feet Intended as Dog Treats: A Pilot Study

**DOI:** 10.1155/vmi/4081691

**Published:** 2026-07-28

**Authors:** Carlotta Marini, Francesco Lipani, Carla Sabia, Mario Nicotra, Ramona Iseppi, Giorgio Biscontini, Matteo Cerquetella, Marco Meschiari, Giuseppe Di Gregorio, Mahmoud Alagawany, Roberta Tardugno, Filomena Corbo, Alessandro Di Cerbo

**Affiliations:** ^1^ School of Biosciences and Veterinary Medicine, University of Camerino, 62024, Matelica, Italy, unicam.it; ^2^ Laboratory of Clinical Chemistry and Microbiology, Hesperia Hospital, 41125, Modena, Italy, hesperia.it; ^3^ Department of Chemical and Geological Sciences, University of Modena and Reggio Emilia, 41125, Modena, Italy, unimore.it; ^4^ Neotron SPA, 41126, Modena, Italy; ^5^ Department of Poultry, Faculty of Agriculture, Zagazig University, Zagazig 44511, Egypt, zu.edu.eg; ^6^ Department of Pharmacy-Drug Sciences, University of Bari “A. Moro”, 70125, Bari, Italy, uniba.it

**Keywords:** antibiotic residues, antimicrobial resistance, dehydrated chicken feet, One Health

## Abstract

The pet population is steadily increasing worldwide, and treats, which account for approximately 15% of the pet food market, are the fastest‐growing segment of the pet food industry. However, the widespread use of antimicrobials in poultry farms may pose a direct risk to pets’ health, leading to the emergence of antimicrobial resistance. For this reason, we sought to assess the possible presence of drug residues and antimicrobial‐resistant bacteria in a limited cohort of 11 dehydrated chicken feet intended as dog treats purchased from the European market. Residues of nicarbazin (64%), 4‐4′ dinitrocarbanilide (64%), semicarbazide (91%), decoquinate (9%), cypermethrin (27%), dichlorvos (9%), pirimiphos‐methyl (36%), piperonyl butoxide (18%), toltrazuril sulfone (9%), doxycycline (9%), lasalocid A (9%), monensin A (9%), and 37 bacterial strains belonging to the *Staphylococcus* genus (*S. warneri*, *S. haemolyticus*, *S. pasteuri*, and *S. intermedius*) were detected among all samples. *S. warneri* species was resistant to oxacillin, erythromycin, clindamycin, teicoplanin, and tetracycline (25%, 25%, 37.5%, 6.25%, and 6.25%, respectively), *S. haemolyticus* was also resistant to erythromycin, clindamycin, teicoplanin, and tetracycline (52.9%, 11.7%, 29.4%, and 35.2%, respectively), while *S. pasteuri* was resistant to both oxacillin and clindamycin (66.6% and 33.3%, respectively). No resistance was detected for *S. intermedius*. The detection of multiple residues further suggests the possibility of cumulative or synergistic toxic effects, especially with prolonged intake. In this sense, a One Health approach to mitigate risks associated with antimicrobial resistance and chemical exposure in pets and, in turn, in humans becomes mandatory.

## 1. Introduction

Worldwide, the pet population is constantly increasing [1]. The global population of household pets exceeds 703 million, a figure expected to rise further in line with the projected growth of the human population, which is projected to reach 10 billion by 2050 [2]. According to the American Veterinary Medical Association, an almost constant increase in the U.S. households holding a pet occurred between 1991 and 2024, with dogs reaching their highest number in 2020 and 2024 (62 and 59.8 million, respectively), while cats showed a slower but nearly regular increase, reaching a peak in 2024 with 42.1 million [3]. A similar trend has been observed in Europe, where about 166 million households—half of the total—own at least one pet, mainly cats (129 million) or dogs (106 million) [4]. These increases were matched by a flourishing of the pet industry in the United States, which reached $138.6 billion in 2022 and exceeded $143.6 billion in 2023, with related expenditures for pet food and treats of $58 billion and $62.7 billion, respectively [5–7]. Beyond the mere use of pet food for nutritional purposes [8, 9], these trends are also driven by humanization attitudes toward companion animals, which are increasingly regarded as family members [10–12].

Treats account for approximately 15% of the pet food market and are produced by nearly all pet food brands, making them the fastest‐growing segment of the industry [13, 14]. They are also popular among pet owners, who use them not only to give treats but also for functional purposes, such as promoting oral health, providing positive reinforcement for good behaviors during training, or reinforcing the emotional bond with their pets [13, 15, 16]. Among treats, dehydrated chicken‐based snacks, such as feet, necks, and wings, are preferred by pet owners for their unique characteristics, including long shelf life, palatability, and high protein content [17]. Nonetheless, the widespread use of antimicrobials in poultry farms can lead to the accumulation of residues in the aforementioned chicken‐derived snacks, thereby accelerating the development of antimicrobial resistance (AMR) and facilitating the transfer of resistant bacteria between animals and humans [18–22]. Similarly, Morgan et al. isolated *Salmonella* spp. from 13 out of 84 (16%) dried treat samples purchased from both local pet shops and online retailers [23]. The isolated strains were attributable to five serotypes (*S. Dublin, S. Derby, S. Infantis, S. Anatum*, and monophasic *S. Typhimurium*), all of which are recognized as potential human pathogens, with 39% of the strains exhibiting AMR. These findings highlight a potential public health concern, particularly given that such products are often handled with bare hands, thereby increasing the likelihood of human contact with contaminating bacteria [24].

Concerning residues, in 2015, Odore et al. compared the concentration of oxytetracycline (OTC) residues in muscles and bones of experimentally treated chicken, showing that even if the muscle residues did not exceed the 100 μg/kg maximum residue limit (MRL), their concentration in bones was far higher, reaching the parts per million (ppm) range [25]. These residues can contaminate pet food, especially chicken‐based kibbles [26], in which bone meal accounts for 20%–30% of the ingredients [26, 27], and human food (e.g., chicken‐ and turkey‐based würstels), where bone and antibiotic residues have recently been detected [28].

OTC, a tetracycline, is among the most widely used antibiotics in farm animals [29]. In poultry, in particular, it is used to control respiratory and gastrointestinal diseases, whose spread is exacerbated by overcrowding and promiscuity [30]. Nevertheless, several in vitro studies have demonstrated its cytotoxicity and the detrimental effects of its residues on bone cells [25, 30–33]. In K562 cells, for example, OTC induced cytoskeletal mechanical changes and impaired mitochondrial function, leading to apoptosis [31]. Similarly, in human peripheral blood mononuclear cells (PBMCs), it exerts pro‐inflammatory and proapoptotic activities, likely due to OTC‐induced DNA and epigenetic modifications, including DNA and histone methylation [30, 33]. Hence, chronic exposure to antibiotic residues may lead to adverse health outcomes, including alterations in the gut microbiota, allergic reactions, and toxicological effects [21, 22, 34]. In animals, the chronic intake of chicken‐based pet food containing antibiotic residues, specifically OTC, has been linked to pathological conditions, primarily cutaneous adverse food reactions (CAFRs) [26, 27].

To control the presence of drug residues in food, the Commission Regulation (EU) No. 37/2010 established MRLs for pharmacologically active substances, including antibiotics, in foodstuffs of animal origin intended for human nutrition [35]. However, no limit is defined for slaughter byproducts employed to produce pet food and treats [36], except for lasalocid, monensin A, nicarbazin, and decoquinate [37].

Given that bones are an integral part of chicken‐based pet treats, this study aims to analyze the presence of chemicals, antibiotics, and antimicrobial‐resistant bacterial strains in a limited cohort of 11 dehydrated chicken feet intended as dog treats from the European market.

## 2. Materials and Methods

### 2.1. Chemicals and Reagents

All reagents and solvents used were of analytical grade or liquid chromatography–mass spectrometry (LC‐MS) grade. Acetonitrile (ACN), methanol, isopropanol, formic acid, trichloroacetic acid, heptafluorobutyric acid (HFBA), hydrochloric acid (HCl, 37%), ammonium acetate, sodium acetate, sodium sulfate anhydrous (Na_2_SO_4_), potassium phosphate monobasic, sodium hydroxide (NaOH), ethylenediaminetetraacetic acid disodium salt (Na_2_EDTA), dithiothreitol (DTT), benzylpenicillinate‐D_7_ potassium salt, d8‐sarafloxacin hydrochloride trihydrate, tetracycline hydrochloride, 4‐epi‐tetracycline hydrochloride, dimethyl sulfoxide (DMSO), acetone, 3‐amino‐5‐(4‐morpholinomethyl)‐oxazolidin‐2‐one‐D5, 3‐amino‐oxazolidin‐2‐one‐D4, semicarbazide‐13C‐15N2 hydrochloride, 1‐amino‐2,4‐imidazolidinedione‐13C3, and n‐hexane were purchased from Fisher Scientific Italia or Merck Life Science S.r.l. (Milan, Italy). MS grade water was obtained using a Milli‐Q water purification system (Millipore, Burlington, MA, USA). Isotopically labeled internal standards, including [^2^H_4_]‐amoxicillin and [^2^H_5_]‐oxacillin, were purchased from Shimadzu Chemistry & Diagnostics (Illkirch‐Graffenstaden, France).

3,5‐Dinitrosalicylic acid‐13C6 hydrazide was purchased from WITEGA Laboratorien Berlin‐Adlershof GmbH (Berlin, Germany). All the analytical reference standards for veterinary drugs including aminoglycosides, amphenicols, nitrofurans, anthelmintics, macrolides, sulfonamides, coccidiostats, beta‐lactams, lincosamides, tetracyclines, quinolones, and nitrofuran metabolites were obtained from Cambridge Isotope Laboratories (Tewksbury, MA, USA), Santa Cruz Biotechnology (Dallas, TX, USA), DBA Compare (Milan, Italy), and Biomol GmbH (Hamburg, Germany).

### 2.2. Samples

Eleven commercial samples of dehydrated chicken feet from different producers were purchased in the European market between September and December 2025. The sample list, including sample numbers, weights, and countries of origin, is presented in Table 1.

**TABLE 1:** Origin and weight of the different dehydrated chicken feet samples.

Sample *n*	Weight (g)	Origin
1	400	Italy
2	200	Italy
3	200	Belgium
4	250	Spain
5	250	Italy
6	200	Poland
7	250	Spain
8	500	Italy
9	700	Germany
10	200	The Netherlands
11	240	Italy

### 2.3. Chemical and Drug Residues Analyzed in Dehydrated Chicken Feet

The presence of chemical and veterinary drug residues in dehydrated chicken feet samples was investigated using multiple analytical methods. A total of 82 compounds, representing a broad range of pharmacological classes, were screened.

The analytes were categorized into 12 groups according to the analytical methodology used, as shown in Table 2.

**TABLE 2:** Analytes and their respective class groupings according to the applied methodology.

Class	Molecule
Aminoglycosides	Kanamycin A
	Paromomycin
	Spectinomycin

Β‐lactam antibiotics and lincosamides	Amoxicillin
	Ampicillin
	Benzylpenicillin
	Phenoxymethylpenicillin (penicillin V)
	Cloxacillin
	Dicloxacillin
	Oxacillin
	Lincomycin

Amphenicols, anthelmintics, nitrofurans, sulfonamides, coccidiostats, macrolides, nitroimidazole	Chloramphenicol
	Thiamphenicol
	Levamisole
	Nitrofurantoin
	Nitrofurazone
	Furazolidone
	Furaltadone
	Nifursol
	Sulfadiazine
	Sulfamethoxazole
	Sulfadoxine
	Sulfathiazole
	Sulfabenzamide
	Sulfachloropyridazine
	Sulfachlorpyrazine
	Sulfadimethoxine
	Sulfamerazine
	Sulfameter
	Sulfamethazine
	Sulfamethizole
	Sulfamethoxypyridazine
	Sulfamonomethoxine
	Sulfamoxole
	Sulfanilamide
	Sulfaphenazole
	Sulfapyridine
	Sulfaquinoxaline
	Sulfisomidine
	Sulfisoxazole
	Monensin A
	Salinomycin
	Lasalocid A
	Narasin
	Maduramicin
	Decoquinate
	Diclazuril
	Nicarbazin
	Robenidine
	Toltrazuril sulfone
	Trimethoprim
	Erythromycin A
	Spiramycin
	Tylosin A
	Tilmicosin
	Neospiramycin
	Metronidazole
	Dimetridazole
	Ronidazole
	Chlorpromazine
	Colchicine
	Dapsone
	Malachite
	Leucomalachite

Nitrofuran metabolites	1‐Aminohydantoin
	3‐Amino‐5‐morpholinomethyl‐oxazolidin‐2‐one
	3‐Amino‐oxazolidin‐2‐one
	Semicarbazide
	3,5‐Dinitrosalicylic acid hydrazide

Quinolones and tetracyclines	Ciprofloxacin
	Enrofloxacin
	Danofloxacin
	Difloxacin
	Flumequine
	Oxolinic acid
	Sarafloxacin
	Tetracycline and 4‐epitetracycline
	Oxytetracycline and 4‐epioxytetracycline
	Doxycycline
	Chlortetracycline and 4‐epichlortetracycline

Each group was analyzed using specific sample preparation protocols and detection techniques suited to the chemical structures of the selected compounds, as described in the following paragraphs.

### 2.4. Extraction, Purification, and Analysis of Aminoglycosides

For the analysis of aminoglycosides in dehydrated chicken feet samples, 2.5 g of the sample was weighed and defatted using 50 mL of *n*‐hexane. The extraction was carried out using a 5% (w/v) trichloroacetic acid solution to a final volume of 200 mL. For purification, solid‐phase extraction (SPE [500 mg–6 mL], Oasis HLB, Waters SpA, Sesto San Giovanni, Italy) was performed using a concentration procedure. A stock mixture of gentamicin sulfate, neomycin B, dihydrostreptomycin sesquisulfate, streptomycin sulfate, spectinomycin, apramycin sulfate salt, kanamycin A disulfate salt hydrate, amprolium hydrochloride, and paromomycin sulfate was prepared in a 1:1 (v/v) ratio in a solution of ACN and 5% trichloroacetic acid to a final concentration of 20 μg/mL (relative to neomycin B). Calibration solutions were prepared by dilution in an 80% solution containing 3% trichloroacetic acid, 1% HFBA, and 20% ACN to obtain concentrations ranging from 0.5 to 75 μg/L (Es. 0.5–1.25 ‐ 2–5 – 12.5–25 – 50–75 μg/L) as a linear regression (*y* = ab + *b*).

The analysis of aminoglycosides was carried out using a 1290 Infinity II LC System (Agilent Technologies Italia SpA, Milan, Italy) coupled to an AB Sciex API 6500 mass spectrometer equipped with a heated electrospray ionization (ESI) source. Chromatographic separation was performed on an ACQUITY Ultra Performance Liquid Chromatography (UPLC) BEH C18 column (Waters SpA, Sesto San Giovanni, Italy; 1.7 μm, 150 mm × 2.1 mm) maintained at 60°C, with an injection volume of 10 μL.

The mobile phases consisted of solvent A: 0.001 M HFBA, 0.1% (w/v) formic acid, and 5% (v/v) ACN in LC‐MS grade water; and solvent B: 0.005 M HFBA, 0.1% (w/v) formic acid, and 20% (v/v) isopropanol in ACN.

Elution was achieved using the following gradient program at a flow rate of 0.5 mL/min: 0–0.2 min, isocratic at 5% B; 0.2–6.2 min, linear increase to 35% B; 6.2–6.3 min, linear increase to 90% B; and 6.3–6.8 min, isocratic at 90% B. From 7.3 to 7.8 min, the flow rate was increased to 0.7 mL/min, maintaining 90% B. The mobile phase was returned to the initial conditions (5% B) at 7.9 min and held until 9.15 min, after which the flow rate was reset to 0.5 mL/min at 8.15 min.

The mass spectrometer operated in positive ionization mode, and aminoglycosides were detected using multiple reaction monitoring (MRM) during MS/MS acquisition. Source parameters were as follows: ion spray voltage: 5500 V, source temperature 400°C, curtain gas (CUR) at 30 psi, ion source gas 1 at 50 psi, ion source gas 2 at 10 psi, and collision gas (CAD) at medium.

### 2.5. Extraction, Purification, and Analysis of Amphenicols, Anthelmintics, Nitrofurans, Coccidiostats, Macrolides, and Sulfonamides

For each analysis, 2.00 g of the dehydrated chicken feet sample was weighed and then added to 20 mL of an antioxidant and chelating solution (ascorbic acid 1000 mg/L, DTT 20 mg/L, disodium EDTA 1000 mg/kg in water) and to 20 mL of ACN. To facilitate the extraction, 2 g of sodium acetate (CH_3_COONa) and 20 g of Na_2_SO_4_ were added to the sample. Internal standards were also added during this step: About 0.2 mL of chloramphenicol at 20 μg/L and 2 mL of a solution containing ronidazole‐D3 and sulfamethazine‐D4 at 500 μg/L were added to the extraction mixture. This resulted in a final concentration of 50 μg/L of the standard mixture in the sample.

Following extraction, the samples were subjected to purification and dispersed in a test tube containing 250 mg of primary–secondary amine (PSA) resin and 750 mg of magnesium sulfate anhydrous.

The chromatographic analysis was carried out using a 1290 Infinity II LC System (Agilent Technologies Italia SpA, Milan, Italy) coupled to an AB Sciex API 6500 mass spectrometer equipped with a heated ESI source. Different columns were used for each drug family: (A) amphenicols and nitrofurans (ACQUITY UPLC HSS T3, Waters SpA, Sesto San Giovanni, Italy; 1.8 μm; 150 mm × 2.1 mm, 40°C), (B) coccidiostats (CORTECS UPLC C18, Waters SpA; 1.6 μm; 100 mm × 1 mm, 40°C), (C) coccidiostat (ACQUITY UPLC CSH C18, Waters SpA; 1.7 μm; 150 mm × 2.1 mm, 60°C), and (D) ACQUITY UPLC HSS T3 (Waters SpA; 1.8 μm; 150 mm × 2.1 mm).

Different chromatographic elution gradients were optimized and applied for each analyte class. For amphenicols and nitrofurans (injection volume: 2 μL), the separation was performed under isocratic conditions of 99% mobile phase A and 1% mobile phase B at a flow rate of 0.400 mL/min, held for 1.5 min. A linear gradient was then applied, reaching 83% A and 17% B at 5.5 min, 59% A and 41% B at 8.5 min, and 58% A and 42% B at 9.5 min. At 9.6 min, a rapid transition to 1% A and 99% B was initiated and maintained until 11.10 min. The flow rate was increased to 0.700 mL/min between 10.60 and 11.25 min. The system was then re‐equilibrated to the initial conditions (99% A, 1% B) at 11.25 min and maintained at 0.400 mL/min for 25 min.

For coccidiostats (injection volume: 2 μL), the initial conditions of 99% A and 1% B were held for 1.0 min at a flow rate of 0.160 mL/min. A gradient was applied to reach 22.5% B at 3.25 min. The mobile phase was shifted to 99% B at 11.00 min and held until 12.80 min, with a temporary increase in flow rate to 0.350 mL/min from 11.90 to 13.05 min. Initial conditions were restored at 13.05 min and maintained until 14.05 min.

A dedicated elution program was used for lasalocid A (injection volume: 2 μL), starting with 70% B at 0.500 mL/min, held for 0.50 min. A gradient to 76.25% B was applied from 3.00 min, followed by a rapid transition to 95% B at 3.10 min, which was maintained until 4.60 min. The flow rate was increased from 4.60 to 4.75 mL/min over 0.750 min and then returned to the initial conditions (70% B) at 5.00 min.

For the simultaneous analysis of amphenicols, anthelmintics, nitrofurans, coccidiostats, macrolides, and sulfonamides (injection volume: 2.5 μL), an isocratic condition of 1% B was held for 1.5 min at a flow rate of 0.400 mL/min. A gradient elution followed, reaching 41% B at 11.50 min, 51% B at 12.50 min, and 71% B at 14.50 min. The mobile phase was then switched to 99% B at 15.90 min, and the flow rate was progressively increased to 0.800 mL/min at 18.80 min. Initial conditions were restored at 18.90 min and maintained until 20.20 min. The applied gradients were optimized to achieve adequate separation, retention, and peak resolution of each analyte class. Mass spectrometric detection for all analyte classes was performed using LC‐MS/MS in MRM mode. The ionization polarity and instrumental settings were optimized according to the chemical nature of the compounds.

For amphenicols and nitrofurans, as well as coccidiostats, the analysis was performed in negative ion mode, while lasalocid A and the combined group of amphenicols, anthelmintics, nitrofurans, coccidiostats, macrolides, and sulfonamides were analyzed in positive ion mode. All methods used a curtain gas set to 30, a collision gas set to 10, and ion source gases 1 and 2 set to 50 and 20, respectively. The ion spray voltage was set to −4500 V for analyses in negative ion mode and +5500 V for those in positive mode. The source temperature was maintained at 400°C for all methods.

### 2.6. Extraction, Purification, and Analysis of Penicillins, β‐Lactam Antibiotics, and Lincosamides

For the analysis of penicillins, β‐lactam antibiotics, and lincosamides in dehydrated chicken feet samples, 1.00 ± 0.01 g of sample was weighed and extracted with 100 mL of ACN buffered with 0.02 M ammonium acetate at pH 6.2.

Calibration solutions were obtained by diluting [^2^H_4_]‐amoxicillin, [^2^H_5_]‐oxacillin, and benzylpenicillinate‐D_7_ in a solution with 30% methanol and 70% 0.1% ascorbic acid in 0.02 M ammonium acetate buffer, pH 6.2 (1:1), with concentrations from 0 to 25 μg/L for each standard (linear regression *y* = ab + *b*). A standard mix containing isotopically labeled internal standards ([^2^H_4_]‐amoxicillin, [^2^H_5_]‐oxacillin, and benzylpenicillinate‐D_7_) was added to the extraction mixture corresponding to a concentration of 50 μg/L in the sample matrix.

The chromatographic analysis was carried out using a 1290 Infinity II LC System (Agilent Technologies Italia SpA, Milan, Italy) coupled to an AB Sciex API 6500 mass spectrometer equipped with a heated ESI source. Chromatographic separation of penicillins, β‐lactam antibiotics, and lincosamides, including amoxicillin, ampicillin, benzylpenicillin, cloxacillin, dicloxacillin, lincomycin, oxacillin, and phenoxymethylpenicillin, was performed using an ACQUITY UPLC HSS T3 column (Waters SpA, 1.8 μm particle size, 150 mm × 2.1 mm i.d.) maintained at 40°C. The injection volume was set to 2 μL. The mobile phase consisted of solvent A (0.1% formic acid in water with 5% ACN) and solvent B (0.1% formic acid in ACN). Separation was carried out using the following gradient elution: From 0.00 to 1.50 min, solvent B was held at 1% with a flow rate of 0.400 mL/min. Between 1.50 and 11.50 min, the concentration of solvent B was gradually increased to 41%. It was further increased to 51% at 12.50 min, then to 71% by 14.50 min. At 14.60 min, solvent B reached 99% and was held at that concentration until 15.60 min. During this phase, the flow rate was increased to 0.800 mL/min between 15.60 and 16.10 min. Solvent B was returned to 1% at 16.20 min, with the flow rate maintained at 0.800 mL/min until 16.25 min. The flow rate was then reduced back to 0.400 mL/min, and re‐equilibration was completed at 17.50 min. Detection of penicillins, β‐lactam antibiotics, and lincosamides was performed by LC‐MS/MS in positive ionization mode, with MRM as the scan type. The ion source was operated under the following optimized conditions: an ion spray voltage of 5500 V and a source temperature of 400°C. The curtain gas was maintained at 30 psi, ion source gas 1 at 50 psi, and ion source gas 2 at 200 psi. The collision gas pressure was set to 10 to ensure effective fragmentation during MS/MS acquisition.

### 2.7. Extraction, Purification, and Analysis of Quinolones and Tetracyclines

For extraction, 2.00 g of the dehydrated chicken feet sample was weighed and treated with two consecutive 40 mL aliquots of freshly prepared McIlvaine buffer (pH 4) containing ethylenediaminetetraacetic acid disodium salt and ascorbic acid at 1000 mg/L. Calibration solutions were obtained by diluting tetracycline hydrochloride, 4‐epi‐tetracycline hydrochloride, d8‐ciprofloxacin, d8‐sarafloxacin, and d5‐enrofloxacin with both a solution of ACN: 0.1% trichloroacetic acid (30%:70%) and a water solution of 0.1% ascorbic acid, with concentrations from 0 to 50 μg/L for each standard (linear regression *y* = ab + *b*). To each sample, 1 mL of a standard mixture at 500 μg/L was added; the mixture was brought to a final volume of 200 mL using a volumetric flask and then purified by SPE ([6 mL–200 mg], Oasis HLB, Waters SpA, Sesto San Giovanni, Italy). The extract was concentrated to dryness under a nitrogen stream, and 1.5 mL of methyl cyanide was added before LC‐MS analysis.

The chromatographic analysis was carried out using a 1290 Infinity II LC System (Agilent Technologies Italia SpA, Milan, Italy) coupled to an AB Sciex API 6500 mass spectrometer equipped with a heated ESI source. An ACQUITY UPLC HSS T3 column (Waters SpA, 1.8 μm; 150 mm × 2.1 mm, 40°C) was used with an injection volume of 2 μL. The mobile phases used were (A) 0.1% formic acid in a 5% ACN solution (v/v) and (B) 0.1% formic acid in ACN (v/v). The elution began with 1% solvent B at a flow rate of 0.4 mL/min from 0 to 1.5 min. Solvent B was then gradually increased to 41% between 1.5 and 11.5 min, with the flow rate maintained at 0.4 mL/min. From 11.5 to 12.5 min, solvent B increased to 51% and continued to rise to 71% by 14.5 min, while the flow rate remained at 0.4 mL/min. A further increase brought solvent B to 99% at 14.6–15.1 min. The flow rate was then increased to 0.8 mL/min between 15.6 and 16.1 min, with solvent B held at 99%. Solvent B was then returned to 1% between 16.2 and 16.25 min, and the flow rate was reduced back to 0.4 mL/min from 16.5 to 17.5 min.

The detector operated in MS/MS mode using MRM in positive mode. Collision gas was set to 10 psi, curtain gas to 30 psi, and ion source gases 1 and 2 to 50 and 200 psi, respectively. The ion spray voltage was 5500 V, and the temperature was maintained at 400°C.

### 2.8. Extraction, Purification, and Analysis of Nitrofuran Metabolites

A 5 g portion of the sample was weighed and spiked with internal standards at 0.2 μg/mL. Following this addition, 20 mL of n‐hexane was used for preliminary treatment. After this pretreatment, 250 mL of 0.125 M hydrochloric acid was added to the mixture. Subsequently, 20 mL of fresh 0.125 M hydrochloric acid and 0.4 mL of the derivatizing solution were added for derivatization. The sample was incubated at 37°C for 16 h to complete the reaction. Each sample was derivatized with 2‐nitrobenzaldehyde for 16 h at 37°C and purified by SPE ([200 mg–6 mL], Nexus Varian, Altmann Analytik GmbH & Co. KG, Munich, Germany). All standards were diluted in methanol to a final concentration of 20 μg/mL and used for calibration.

For the analysis of nitrofuran metabolites (1‐aminohydantoin, 3‐amino‐5‐morpholinomethyl‐2‐oxazolidinone, 3‐amino‐2‐oxazolidinone, and semicarbazide) and the nitrofuran metabolite (3,5‐dinitrosalicylic acid hydrazide), chromatographic separation was performed using an ACQUITY UPLC BEH C18 column (Waters SpA, Sesto San Giovanni, Italy; 1.7 μm, 150 mm × 2.1 mm) maintained at 60°C, and a 10 μL injection volume was used. The mobile phases used were (A) 0.002 M ammonium formate buffer and (B) 0.25% formic acid in ACN (v/v). The mobile phase initially consisted of 10% phase B, at a flow rate of 0.5 mL/min. This composition was maintained until 0.50 min. From 0.50 to 5.50 min, the mobile phase was held at 20% B. At 5.60 min, the gradient rapidly changed to 99% B and was held until 6.70 min. From 6.70 to 7.20 min, the flow rate was increased to 0.8 mL/min. At 7.30 min, the initial conditions were restored and maintained at 0.5 mL/min until 7.50 min.

For the nitrofuran metabolite, a 5 μL injection volume was used. The initial mobile phase consisted of 55% A and 45% B at a flow rate of 0.5 mL/min and was maintained until 3.00 min. At 3.10 min, the composition changed to 99% B and was held at that value until 4.60 min. Between 4.10 and 4.60 min, the flow rate was increased to 0.8 mL/min. The mobile phase was returned to 45% B at 4.70 min and maintained at that level until 6.00 min, with the flow rate reduced to 0.5 mL/min at 4.90 min.

Detection was performed using LC‐MS/MS in MRM mode. Nitrofuran metabolites, including 1‐aminohydantoin, 3‐amino‐5‐morpholinomethyl‐2‐oxazolidinone, 3‐amino‐2‐oxazolidinone, and semicarbazide, were analyzed in positive ionization mode, while 3,5‐dinitrosalicylic acid hydrazide was analyzed in negative ionization mode. Source parameters included a curtain gas at 30 psi, collision gas at 10 psi, ion source gas 1 at 50 psi, and ion source gas 2 at 10 psi. The ion spray voltage was set to 5300 V for nitrofuran metabolites, except for 3,5‐dinitrosalicylic acid hydrazide, which was set to 4500 V. The source temperature was maintained at 400°C for all the metabolites.

### 2.9. Isolation and Identification of Bacterial Strains

Twenty‐five grams of dehydrated chicken feet was placed in 225 mL Tryptic Soy Broth (TSB bioMérieux, Florence, Italy) and incubated for 24 h at 37°C. After incubation, the broths were homogenized for 2 min in sterile plastic bags in a Stomacher (Lab Blender, Seward, London, UK).

For each sample, one plate of MacConkey agar (bioMérieux, Florence, Italy) and one of Mannitol salt agar (bioMérieux, Florence, Italy) were used to investigate the presence of *Enterobacteriaceae* and *Staphylococci,* respectively. In particular, 100 μL of each appropriate dilution was spread onto each plate and incubated at 37°C for 24 h. Where possible, up to 3 colonies from each plate were selected and subcultured onto the same medium again at 37°C for 24 h. Species were identified using the VITEK 2 system (Card GP‐659, bioMérieux, Florence, Italy).

### 2.10. Antimicrobial Susceptibility Test (AST)

The isolates were tested for antimicrobial susceptibility using an automated system (VITEK 2, Card AST‐659, bioMérieux) according to the manufacturer’s instructions. Briefly, three to five colonies of *Staphylococcus* spp., with a concentration of approximately 0.5 McFarland, were suspended in sterile 0.45% NaCl solution. The suspension was then loaded onto the card and incubated in the VITEK 2 system for 5–8 h. Antibiotics tested included oxacillin, clindamycin, erythromycin, gentamicin, levofloxacin, linezolid, daptomycin, rifampicin, tetracycline, fusidic acid, trimethoprim/sulfamethoxazole, tigecycline, and glycopeptides (teicoplanin, vancomycin). VITEK 2, an automated identification/antibiotic susceptibility test (ID/AST) analyzer, uses the advanced expert system (AES) that integrates EUCAST expert and breakpoint documents (European Committee on Antimicrobial Susceptibility Testing, EUCAST 2023).

The minimum inhibitory concentrations (MICs) of *doxycycline* for *Staphylococci* were determined by broth microdilution in Mueller–Hinton broth (bioMérieux, Florence, Italy), with concentrations ranging from 0.125 to 4 μg/L. This method was performed in accordance with the EUCAST 2025 guidelines, using EUCAST clinical breakpoints. Quality control was performed in accordance with the EUCAST 2025 guidelines using *Staphylococcus aureus* ATCC 29213.

### 2.11. Statistical Analysis

Data were analyzed using GraphPad Prism 9 software (GraphPad Software, Inc., La Jolla, CA, USA) and expressed as mean ± standard deviation (SD). A descriptive statistic was applied to all drugs, metabolite residues, and resistant strains.

## 3. Results

### 3.1. Drug Residue Detection

In Table 3, drug and metabolite residues detected in dehydrated chicken feet, along with MRLs for undesirable substances in feed materials [37], are reported along with their concentrations.

**TABLE 3:** Drug and metabolite residue concentrations detected in dehydrated chicken feet.

Sample #	Drug	Result ± SD	Recovery %	Measurement unit	LQ	MRL (Dir. 2002/32/CE)
1	Nicarbazin	10 ± 5	101	μg/kg	5	1.25 mg/kg
	4‐4′ Dinitrocarbanilide	7.2 ± 3.5	101	μg/kg	5	
	Semicarbazide	2.5 ± 0.7	99	μg/kg	0.5	

2	Semicarbazide	1.8 ± 0.7	99	μg/kg	0.5	

3	Decoquinate	230 ± 101	110	μg/kg	5	0.4 mg/kg
	Nicarbazin	180 ± 79	101	μg/kg	5	
	4‐4′ Dinitrocarbanilide	120 ± 53	101	μg/kg	5	
	Semicarbazide	510 ± 143	99	μg/kg	0.5	
	Cypermethrin, including other mixtures of constituent isomers (sum of isomers)	0.093 ± 0.041	95	mg/kg	0.05	
	Dichlorvos	0.065 ± 0.029	95	mg/kg	0.05	
	Pirimiphos‐methyl	0.11 ± 0.05	96	mg/kg	0.05	

4	Nicarbazin	17 ± 7	101	μg/kg	5	1.25 mg/kg
	4‐4′ Dinitrocarbanilide	12 ± 5	101	μg/kg	5	
	Semicarbazide	1.5 ± 0.7	99	μg/kg	0.5	
	Piperonyl butoxide	0.080 ± 0.035	96	mg/kg	0.05	
	Pirimiphos‐methyl	0.18 ± 0.07	96	mg/kg	0.05	

5	Nicarbazin	36 ± 16	101	μg/kg	5	1.25 mg/kg
	4‐4′ Dinitrocarbanilide	25 ± 11	101	μg/kg	5	
	Semicarbazide	21 ± 6	99	μg/kg	0.5	

6	Nicarbazin	37 ± 16	101	μg/kg	5	1.25 mg/kg
	4‐4′ Dinitrocarbanilide	26 ± 11	101	μg/kg	5	
	Semicarbazide	2.2 ± 0.6	99	μg/kg	0.5	
	Pirimiphos‐methyl	0.31 ± 0.12	96	mg/kg	0.05	

7	Semicarbazide	2.6 ± 0.7	99	μg/kg	0.5	
	Cypermethrin, including other mixtures of constituent isomers (sum of isomers)	0.11 ± 0.05	95	mg/kg	0.05	

8	Semicarbazide	89 ± 25	99	μg/kg	0.5	
	Cypermethrin, including other mixtures of constituent isomers (sum of isomers)	0.13 ± 0.06	95	mg/kg	0.05	
	Piperonyl butoxide	0.62 ± 0.21	96	mg/kg	0.05	

9	Semicarbazide	0.6 ± 0.26	99	μg/kg	0.5	
	Toltrazuril sulfone	32 ± 15	99	μg/kg	25	
	Pirimiphos‐methyl	0.75 ± 0.25	96	mg/kg	0.05	

10	Nicarbazin	31 ± 14	101	μg/kg	5	1.25 mg/kg
	4‐4′ Dinitrocarbanilide	22 ± 10	101	μg/kg	5	
	Semicarbazide	530 ± 148	99	μg/kg	0.5	

11	Doxycycline	90.5 ± 40	101	μg/kg	50	
	Lasalocid A	8 ± 3.5	102.2	μg/kg	5.0	1.25 mg/kg
	Monensin A	25.95 ± 11.4	88	μg/kg	5.0	1.25 mg/kg
	Nicarbazin	5 ± 2.5	101	μg/kg	5.0	1.25 mg/kg
	4‐4′ Dinitrocarbanilide	3.6 ± 1.7	101	μg/kg	5.0	

**FIGURE 1:**
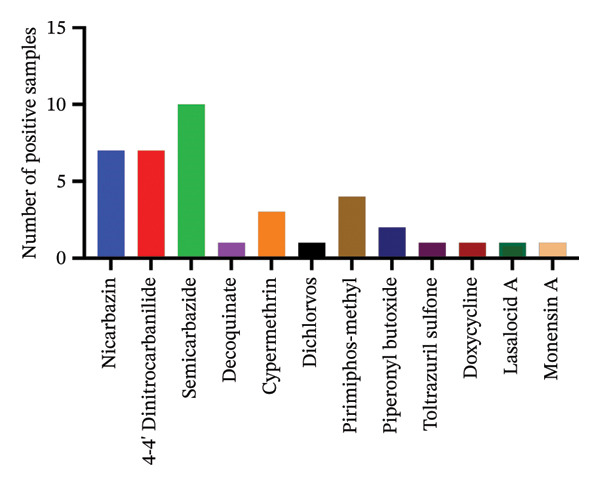
Graphical representation of the prevalence of drug and metabolite residues.

The prevalence of each drug and its metabolite residues is shown in Figure 1.

### 3.2. Isolation and Identification of Bacterial Strains

**FIGURE 2:**
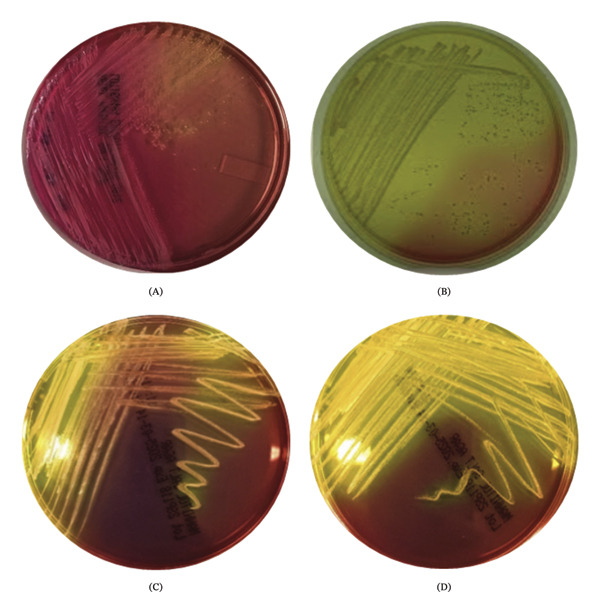
Pictures of (A) *S. intermedius*, (B) *S. haemolyticus,* (C) *S. pasteuri*, and (D) *S. warneri*.

A total of 37 bacterial strains belonging to the *Staphylococcus* genus were isolated from almost all samples, except for #3, #7, and #9, and were identified as *S. warneri* (16), *S. haemolyticus* (17), *S. pasteuri* (3), and *S. intermedius* (1) using the VITEK 2 system (Figure 2).

Table 4 shows the percentages of AMR detected in *Staphylococcus* isolates against 14 antimicrobials, as determined by the VITEK 2 system (Card AST‐GP‐659, bioMérieux, Florence, Italy).

**TABLE 4:** Antibiotic resistance rates (%) in *Staphylococcus* strains isolated.

Antibiotic	Strains			
	*S. warneri* (*n* = 16)	*S. haemolyticus* (*n* = 17)	*S. intermedius* (*n* = 1)	*S. pasteuri* (*n* = 3)
Oxacillin	25	0	0	66.6
Gentamicin	0	0	0	0
Levofloxacin	0	0	0	0
Erythromycin	25	52.9	0	0
Clindamycin	37.5	11.7	0	33.3
Linezolid	0	0	0	0
Daptomycin	0	0	0	0
Teicoplanin	6.25	29.4	0	0
Vancomycin	0	0	0	0
Tetracycline	6.25	35.2	0	0
Tigecycline	0	0	0	0
Fusidic acid	0	0	0	0
Rifampicin	0	0	0	0
Trimethoprim‐sulfamethoxazole	0	0	0	0

*S. warneri* was resistant to oxacillin, erythromycin, clindamycin, teicoplanin, and tetracycline (25%, 25%, 37.5%, 6.25%, and 6.25%, respectively). The results also showed resistance of *S. haemolyticus* to erythromycin, clindamycin, teicoplanin, and tetracycline (52.9%, 11.7%, 29.4%, and 35.2%, respectively). Moreover, *S. pausteri* was resistant to both oxacillin (66.6%) and clindamycin (33.3%), while *S. intermedius* exhibited sensitivity to all the tested antibiotics.

All staphylococcal isolates were susceptible to gentamicin, levofloxacin, linezolid, daptomycin, vancomycin, tigecycline, fusidic acid, rifampicin, and trimethoprim/sulfamethoxazole.

In Table 5, MIC values for doxycycline are reported by the broth microdilution method.

**TABLE 5:** MIC for doxycycline of the 37 strains.

Strains	MIC (μg/mL)	BP doxycycline *S* ≤ 0.25‐*R* > 1
(*n* = 13) *S. warneri*	0.125	S
(*n* = 2) *S. warneri*	2	R
(*n* = 1) *S. warneri*	1	S
(*n* = 3) *S. pasteuri*	0.125	S
(*n* = 6) *S. haemolyticus*	2	R
(*n* = 11) *S. haemolyticus*	0.125	S
(*n* = 1) *S. intermedius*	0.125	S

Eight of 37 (21.6%) *Staphylococci* were resistant (MIC = 2 μg/mL), 28 of 37 (75.7%) were sensitive (MIC = 0.125 μg/mL), and 1 out of 37 (2.7%) was sensitive (MIC = 1 μg/mL).

## 4. Discussion

In this pilot study, we conducted a proof‐of‐concept investigation based on previous observations regarding the detection of drug residues, particularly OTC, in chicken‐based pet food [26, 27]. Although the concentration of this drug resulted below the MRLs [35], we observed that cats and dogs receiving such pet food daily exhibited clinical signs, including drooling, intense itching of the back and neck, neck eczema, chronic conjunctivitis, stomatitis, otitis, diarrhea, generalized anxiety, and dermatitis [26, 27]. Interestingly, after substituting the diet with a chicken‐free formulation, all the aforementioned symptoms showed a significant reduction, coinciding with a decrease in serum OTC concentrations. Therefore, we conducted in vitro experiments to assess the potential role of OTC in the onset of these clinical manifestations, demonstrating its pro‐inflammatory, pro‐apoptotic, and genotoxic activity even at concentrations below the MRLs [29–31, 33, 38–45].

Moreover, a study by Odore et al. [25] conducted on 72 one‐day‐old broiler chickens treated with OTC in compliance with regulations governing dose concentration, treatment duration, and withdrawal times [46] confirmed the presence of the antibiotic in animal muscle tissue, although below the MRLs (12.3 ± 6.9 μg/kg). However, the study revealed a markedly higher accumulation in bone tissue (1286.3 ± 256.6 g/kg), far exceeding the MRLs.

In light of these observations and the entry into force of Regulation (EU) 2019/6 on veterinary medicinal products [47], we aimed at reassessing the possible presence of drug residues, including OTC, in a limited cohort of 11 dehydrated chicken feet intended as dog treats purchased from the European market, in which the bone matrix represented the most abundant component.

Thirty‐seven *Staphylococci* belonging to *warneri* (16)*, haemolyticus* (17)*, intermedius* (1), and *pasteuri* (3) species, showing different resistance rates to oxacillin, erythromycin, clindamycin, teicoplanin, and tetracycline, were isolated from almost all samples, except for #3, #7, and #9. Notably, 2 *S. warneri* strains and 6 *S. haemolyticus* strains were isolated from sample #11, the only one with a high doxycycline concentration (90.5 ± 40 g/kg), and were resistant to this antibiotic. This finding raises serious concerns regarding the possible horizontal gene transfer [48] of doxycycline resistance genes following ingestion of the treatment by the animals, with consequent onset of antibiotic resistance phenomena, spread of resistant bacteria through the feces, and possible environmental contamination, including humans, thereby representing a One Health risk [49]. Several studies have suggested that companion animals can transmit or share resistance genes and/or resistant bacteria with their owners [50–56], with carbapenemase‐producing *Enterobacteriaceae,* extended‐spectrum β‐lactamase‐producing Gram‐negative bacteria, methicillin‐resistant *S. aureus,* and vancomycin‐resistant *enterococci* among the most frequently detected microorganisms [56, 57]. In a recent study by Zhao et al., the authors reported the presence of 18 tetracycline resistance genes, including *tetX,* in the gut microbiota of dogs and their owners [55], further supporting the close relationship between humans and companion animals and corroborating our hypothesis.

The absence of *Enterobacteriaceae* and the isolation of 37 *Staphylococcus* spp. strains from almost all samples, particularly *S. haemolyticus*, *S. warneri*, and *S. intermedius*, are noteworthy. Some of these species harbor clinically relevant resistance genes and may act as reservoirs for horizontal gene transfer, even outside clinical settings [58]. The detection of multidrug‐resistant strains, with notable resistance to macrolides (erythromycin), lincosamides (clindamycin), and glycopeptides (teicoplanin), raises concerns about the microbial safety of pet food products and their potential role in the dissemination of AMR.

Although all isolates were susceptible to key antibiotics, such as linezolid, vancomycin, daptomycin, and tigecycline, agents typically reserved for severe human infections, the presence of doxycycline resistance (MIC = 2 μg/mL in 21.6% of isolates) indicates early resistance development and selective pressure. This phenomenon may result from subtherapeutic exposure during production or from inadequate cooking processes that fail to eliminate resistant microorganisms [59], particularly given the increasing global use of antimicrobials in food‐producing animals [60]. The widespread use of veterinary antimicrobials represents a major driver of AMR, posing serious risks to animal and human health through potential transmission pathways. Effective global monitoring of antimicrobial consumption is therefore essential to assess progress in reducing the agricultural dependence on antimicrobials and to identify countries where targeted stewardship interventions are most urgently needed [61].

In addition to doxycycline and resistant bacteria, 1 out of 11 samples (#11) also showed the presence of three coccidiostats, lasalocid A (8 ± 3.5 μg/kg), nicarbazin (5 ± 2.5 μg/kg), along with its marker residue 4,4′‐dinitrocarbanilide (3.6 ± 1.7) [62], and monensin A (25.95 ± 11.4 μg/kg). Although ionophore antibiotics are widely recognized as the most frequently detected residues in poultry‐derived products in European monitoring systems [63, 64], the observed concentrations may reflect therapeutic treatment for *Eimeria* infection [65] rather than noncompliance with withdrawal periods. Monensin A is commonly marketed in combination with nicarbazin (Monimax) and is considered safe for chickens at a maximum inclusion level of 50 mg/kg feed for each compound [66]. Furthermore, the no observed effect levels (NOELs) identified for monensin sodium and 4,4′‐dinitrocarbanilide, 0.3 and 20 mg/kg·bw/day, respectively, are far above the concentrations detected in our samples [66]. Similarly, lasalocid A and monensin A levels were well below the estimated dietary NOEL of 1–2.5 mg/kg·bw/day for dogs [67].

Nicarbazin and 4,4′‐dinitrocarbanilide were also detected in 7 out of 11 samples (#1, #3, #4, #5, #6, #10, and #11), with concentrations ranging from 5 ± 2.5 μg/kg to 180 ± 79 μg/kg for nicarbazin and from 3.6 ± 1.7 μg/kg to 120 ± 53 μg/kg for 4,4′‐dinitrocarbanilide.

Surprisingly, 10 out of 11 samples (#1–10) contained semicarbazide, with concentrations ranging from 0.6 ± 0.26 μg/kg to 530 ± 148 μg/kg. Semicarbazide is a metabolite of the nitrofurazone, a nitrofuran antibiotic banned in the EU due to its carcinogenic properties [68] and therefore listed in Table 2 of Regulation (EU) 37/2010 [35]. Although semicarbazide is commonly used as a marker for nitrofuran misuse in food‐producing animals, it may also originate from azodicarbonamide used as a food additive [69, 70] or from hypochlorite food treatment [71, 72]. To date, reports of semicarbazide residues have primarily focused on dairy protein ingredients [73], fishery products [74, 75], and flour [76]. Toxicological studies indicate that semicarbazide can inhibit semicarbazide‐sensitive amine oxidases [77], lysyl oxidase [78], and glutamic acid decarboxylase [79] with potential effects on glucose regulation, endothelial function, and neurologic activity, as well as anti‐estrogenic [80] and anti‐androgenic [81] properties. Nevertheless, the highest concentration detected was well below the NOEL established in male (0.6 mg/kg/day) and female (3.9 mg/kg/day) rats [82].

Further analysis revealed that 1 out of 11 samples (#3) contained decoquinate (230 ± 101 μg/kg) and dichlorvos (0.065 ± 0.029 mg/kg). Decoquinate, when administered to broiler chickens at 40 mg/kg, is neither genotoxic nor carcinogenic, and does not require MRLs [83]. The concentration detected was approximately 200‐fold below the established safe level.

Regarding dichlorvos, the concentration was nearly 100 times lower than the NOEL for cholinergic effects in dogs (1.0 mg/kg‐day) [84], and below levels associated with biochemical alterations in poultry‐fed contaminated feed [85].

Cypermethrin and its isomers were detected in 3 out of 11 samples (#3, #7, and #8) at concentrations ranging from 0.093 ± 0.041 mg/kg to 0.13 ± 0.06 mg/kg, approximately 100 times higher than levels reported in chicken samples from the Brazilian market [86]. Cypermethrin is a synthetic pyrethroid widely used to control ectoparasites in livestock and companion animals [87–89]. MRLs for cypermethrin isomers in poultry tissues have been established at 0.1 mg/kg for muscle and fat and 0.05 mg/kg for liver [35, 90]. The NOEL for α‐cypermethrin in dogs is 1.5 mg/kg·bw/day, with an ADI of 0–0.02 mg/kg bw [91]. Consequently, prolonged intake could raise concerns, particularly given the possible additive genotoxicity [92].

Low cypermethrin concentrations may also result from contamination of poultry litter, commonly associated with ectoparasite control practices [93]. Supporting this hypothesis, Neskovic et al. demonstrated altered liver enzyme activity in chickens fed cypermethrin, without tissue accumulation [94].

Four out of 11 samples (#3, #4, #6, and #9) also contained pirimiphos‐methyl (0.11 ± 0.05 mg/kg to 0.75 ± 0.25 mg/kg). Although MRLs for poultry tissues remain at 0.01 mg/kg [95], long‐term exposure risks have been identified [96]. Despite in vitro evidence indicating cytotoxicity only at much higher concentrations [97], the detected levels exceed the ADI for dogs (0.004 mg/kg·bw/d) [98], raising concerns about chronic exposure.

One out of 11 samples (#8) showed the concomitant presence of cypermethrin and the synergist piperonyl butoxide (0.62 ± 0.21 mg/kg). Residue levels were consistent with those reported following dermal exposure of poultry to 10 or 100 mg/kg for 5 days (0.006–1.2 mg/kg) [99].

Although MRLs have not been set for pet food, in 2002, the FAO calculated maximum and mean intakes of piperonyl butoxide for poultry at feed concentrations of 99.3 and 27.4 mg/kg, respectively [99]. For the maximum intake, values ranged from < 0.08 mg/kg in liver to 0.38 mg/kg in muscle to 5.6 mg/kg in fat, and mean intake values ranged from < 0.01 mg/kg in liver to 0.058 mg/kg in muscle to 0.52 mg/kg in fat. Moreover, residues in poultry products from dermally treated poultry ranged from 0.26 mg/kg in liver to 3.8 mg/kg in skin, 1 mg/kg in muscle, and 2 mg/kg in fat. Our results are in line with the aforementioned findings and those reported in laying hens, suggesting possible contamination following topical application. In 2011, the FAO also calculated both NOAEL (14.8 mg/kg·bw/d) and LOAEL (63 and 61 mg/kg·bw/d, in male and female dogs, respectively) in dogs following an acute exposure for 8 weeks at 0, 500, 1000, 2000, and 3000 mg/kg of piperonyl butoxide in their diet, and at 0, 14.7, 32, 63, and 90 mg/kg·bw/d and 0, 14.8, 37, 61, and 85 mg/kg·bw/d in males and females, respectively [100]. In light of these toxicological data, further studies are needed to evaluate the potential cumulative effects of prolonged intake of our sample in dogs.

Finally, 1 out of 11 samples (#9) contained toltrazuril sulfone (32 ± 15 μg/kg), a marker for toltrazuril use in livestock [35, 101]. Such a concentration was 20 times higher than the NOAEL proposed for dogs, i.e., 1.5 mg/kg·bw/d [102], and was also higher than that of ethanamizuril (e.g., 60 mg/kg), a novel triazine compound [103]. Along with the aforementioned toxicity data, Zhang et al. reported a significant toxicity of toltrazuril also in DF‐1 cells treated with 40, 60, 80, 200, and 300 μg/mL of the drug [104], thereby allowing us to hypothesize a further additive cytotoxic effect exerted by a prolonged intake of the sample.

## 5. Study Limitation

Notably, this research has some study limitations. In this regard, the limited sample cohort and the limited geographical representation across European countries are the main relevant limitations. Therefore, an extension to a broader sample cohort that more accurately represents all European countries is needed to clearly depict the actual situation regarding the presence of drug and metabolite residues and resistant strains in dehydrated chicken feet intended as dog treats.

## 6. Conclusions

This pilot study highlights the potential health risks posed by long‐term, chronic low‐dose exposure to drug residues, including antibiotics, antiparasitics, and insecticides, in dehydrated chicken feet intended as dog treats. Of particular concern was the presence of semicarbazide, a banned nitrofuran metabolite, and multidrug‐resistant *Staphylococcus* species exhibiting doxycycline resistance, which poses risks to animal and human health through the potential transmission of AMR. The detection of multiple residues further suggests the possibility of cumulative or synergistic toxic effects, especially with prolonged intake. Moreover, along with the lack of EU MRLs for slaughter byproducts intended for pet feeding, these findings depict a delicate scenario in which the need for stricter surveillance, improved residue control in animal‐derived pet products, and the adoption of a One Health approach to mitigate risks associated with AMR and chemical exposure becomes mandatory.

## Author Contributions

Carlotta Marini: software, validation, formal analysis, resources, writing–original draft preparation, writing–review and editing, visualization, project administration, and funding acquisition.

Francesco Lipani: methodology, investigation, writing–original draft preparation, and writing–review and editing.

Carla Sabia: conceptualization, data curation, writing–original draft preparation, writing–review and editing, and visualization.

Mario Nicotra: software, formal analysis, investigation, writing–original draft preparation, and writing–review and editing.

Ramona Iseppi: formal analysis, investigation, writing–original draft preparation, and writing–review and editing.

Giorgio Biscontini: methodology, validation, resources, writing–original draft preparation, and writing–review and editing.

Matteo Cerquetella: formal analysis, data curation, writing–original draft preparation, and writing–review and editing.

Marco Meschiari: methodology, investigation, writing–original draft preparation, and writing–review and editing.

Giuseppe Di Gregorio: methodology, investigation, writing–original draft preparation, and writing–review and editing.

Mahmoud Alagawany: software, formal analysis, investigation, writing–original draft, and writing–review and editing.

Roberta Tardugno: validation, data curation, writing–original draft preparation, and writing–review and editing.

Filomena Corbo: Validation, writing–original draft preparation, writing–review and editing, and visualization.

Alessandro Di Cerbo: conceptualization, validation, resources, data curation, writing–original draft preparation, writing–review and editing, visualization, supervision, project administration, and funding acquisition.

## Funding

This research received no external funding. Open access publishing facilitated by Universita degli Studi di Camerino, as part of the Wiley ‐ CRUI‐CARE agreement.

## Disclosure

All authors have read and agreed to the published version of the manuscript.

## Conflicts of Interest

The authors declare no conflicts of interest.

## Data Availability

The original contributions presented are included in the article. Further inquiries can be directed to the corresponding author.
